# Improved scFv Anti-HIV-1 p17 Binding Affinity Guided from the Theoretical Calculation of Pairwise Decomposition Energies and Computational Alanine Scanning

**DOI:** 10.1155/2013/713585

**Published:** 2013-11-07

**Authors:** Panthip Tue-ngeun, Kanchanok Kodchakorn, Piyarat Nimmanpipug, Narin Lawan, Sawitree Nangola, Chatchai Tayapiwatana, Noorsaadah Abdul Rahman, Sharifuddin Md. Zain, Vannajan Sanghiran Lee

**Affiliations:** ^1^Computational Simulation Modelling Laboratory (CSML), Department of Chemistry and Center of Excellence for Innovation in Chemistry and Materials Science Research Center, Faculty of Science, Chiang Mai University, Chiang Mai 50200, Thailand; ^2^Thailand Center of Excellence in Physics, Commission on Higher Education, 328 Sri Ayutthaya Road, Bangkok 10400, Thailand; ^3^Department of Medical Technology, School of Allied Health Sciences, University of Phayao, Phayao 56000, Thailand; ^4^Division of Clinical Immunology, Department of Medical Technology, Faculty of Associated Medical Sciences, Chiang Mai University, Chiang Mai 50200, Thailand; ^5^Biomedical Technology Research Unit, National Center for Genetic Engineering and Biotechnology, National Science and Technology Development Agency, Faculty of Associated Medical Sciences, Chiang Mai University, Chiang Mai 50200, Thailand; ^6^Department of Chemistry, Faculty of Science, University of Malaya, 50603 Kuala Lumpur, Malaysia

## Abstract

Computational approaches have been used to evaluate and define important residues for protein-protein interactions, especially antigen-antibody complexes. In our previous study, pairwise decomposition of residue interaction energies of single chain Fv with HIV-1 p17 epitope variants has indicated the key specific residues in the complementary determining regions (CDRs) of scFv anti-p17. In this present investigation in order to determine whether a specific side chain group of residue in CDRs plays an important role in bioactivity, computational alanine scanning has been applied. Molecular dynamics simulations were done with several complexes of original scFv anti-p17 and scFv anti-p17mutants with HIV-1 p17 epitope variants with a production run up to 10 ns. With the combination of pairwise decomposition residue interaction and alanine scanning calculations, the point mutation has been initially selected at the position MET100 to improve the residue binding affinity. The calculated docking interaction energy between a single mutation from methionine to either arginine or glycine has shown the improved binding affinity, contributed from the electrostatic interaction with the negative favorably interaction energy, compared to the wild type. Theoretical calculations agreed well with the results from the peptide ELISA results.

## 1. Introduction

One of the challenges in molecular biology consists in improving the structural, functional properties or binding activities of proteins. The antibodies constitute an excellent model to test the potential approaches to this problem because they constitute a homogeneous family of proteins and a large amount of structural and functional data is available. The antigen-binding sites of immunoglobulins are embedded into the variable heavy and light chain domains (*V*_*H*_, *V*_*L*_) and are specially separated from the effector function-mediating regions located in the Fc fragment. One type of genetically engineered antibody is the single chain Fv fragment (scFv). Single chain Fv fragments are genetically engineered polypeptides that contain a heavy chain variable region (VH) linked to a light chain variable region (VL) via a flexible peptide linker. Each VH and VL domain contains three complementarity determining regions (CDRs). CDRs are short amino acid sequences that vary greatly among antibody molecules and, thus, are responsible for generating the great diversity of antibody binding specificity. The combination of the CDRs of the VH plus the CDRs of the VL determines the binding specificity of any given antibody. Single chain Fv fragments display the binding specificity, monovalent binding affinity of full-size antibodies, and provide the added benefit of relative ease of genetic manipulation and expression. Given their advantages of small size and antigen specificity encompassed within a single polypeptide chain, scFvs are the most common type of recombinant antibody fragment used for intracellular antibody expression [1].

Several computation approaches have been applied to study the protein-ligand interaction by the combination of computational alanine scanning and free energy decomposition methods. The molecular mechanics poisson-Boltzmann surface area (MMPBSA) [2] and molecular mechanics generalized born surface area (MMGBSA) [3] approaches are used to evaluate binding free energy in the computational alanine scanning. Both PB and GB implicit solvent models were used to calculate the energy contribution of each residue in the binding free energy on a decomposition basis. The results from computational alanine scanning and free energy decomposition methods indicated the important residues for binding 4 of lipopeptide inhibitor to *E*. *coli* SPase [4] and recognize the key residues in the ATP binding site of GyrB subunit from *Escherichia coli* bound with the inhibitors clorobiocin, novobiocin, and 5′-adenylyl-*β*-*γ*-imidodiphosphate [5].

In our previous work, scFv anti-p17 was simulated based on molecular modeling of its homologue structure. The antibody-antigen complex models were generated using the molecular docking that predicted the most favorable binding interaction. Then the interactions between nine peptide epitopes and the scFv anti-p17 in water were analyzed using molecular dynamics (MDs) simulation to evaluate the binding free energy and pairwise decomposition or residue-based energy calculation of complexes in solution using the molecular mechanics/poisson-Boltzmann surface area (MM-PBSA) and molecular mechanics/generalized born surface area (MM-GBSA) methods. The latter analysis can provide interesting information in terms of electrostatic and van der Waals energies, solvation energies, and entropic contributions at the binding interface. Pairwise decomposition of residue interaction energies of the complexes between scFv anti-p17 and its variants have indicated that the specific residues located in the complementary determining regions (CDRs) of scFv anti-p17, MET100, LYS101, ASN169, HIS228, and LEU229 play a crucial role in the effective binding interaction [6].

To determine whether the side chain of the specific residue in CDRs of scFv anti-p17 plays an important role in bioactivity, computational alanine scanning was carried out in this study. Computational alanine scanning uses a simple free energy function to calculate the effects of alanine mutations on the binding free energy of a protein-protein complex. It involves the free energy decomposition involving MM-PBSA method [2, 3, 7–11] and MM-GBSA method [3, 12] to investigate the binding modes in detail at the atomic level and also to estimate protein stabilities [13]. The input for the computational alanine scanning consisted of a three-dimensional modeled structure of the scFv anti-p17. Potential amino acids that involved changing nonalanine amino acids into alanines will be listed up. The relevant change of more than 1 kcal mol^−1^ in decomposition energy calculation indicated the important residues involving in the binding [14].

In this study, theoretical modeling and molecular dynamics simulations investigation of scFv against HIV-1 epitope at C-terminal on p17 (scFv anti-p17) has been performed to specify the key residues in the binding. From the search in National Center for Biotechnology Information (NCBI) database, 9 different variants of HIV-1 epitope at C-terminal which show different binding activities upon binding with scFv were found. Computational alanine scanning was utilized to investigate the effect of side chain atoms of the residues in CDR loops of scFv anti-p17. With the combination of the two techniques, the selected point mutation to improve the binding affinity between the scFv anti-p17 and its variants was identified. These residues were mutated to improve the binding activities guided from statistically preferred substitutions observed in buried residues and exposed residues due to their solvent exposed area and side chain charge which generally correlate with side chain physicochemical properties [15]. Finally, as validated by the experimental results, we have designed new scFv anti-p17 which binds better with HIV-1 epitope at C-terminal on p17.

## 2. Materials and Methods

### 2.1. Molecular Docking

The built model reported in the previous work [6] of primary sequence of the scFv anti-p17 protein obtained by Tewari [16] was used in this study. The general docking protocol and potential functions employed in CDOCKER have been described in prior articles [17]. In this work, docking of the peptides to scFv anti-p17 was conducted using CDOCKER. CDOCKER is a grid-based molecular docking method that employs CHARMm. The receptor is held rigid while the ligands are allowed to flex during the refinement. Ligands are assumed to have already been roughly docked into the receptor binding site. The active site pocket of the receptor was found on the CDRs of scFv anti-p17 by the Discovery Studio 2.0 (Accelrys Software Inc.). A site sphere radius of 25 Å was set to assign the binding pocket, and the ligand partial charge method for assigning partial charges to the ligands during force field assignment was CHARMm. Other parameters were set as default. The lowest docking interaction structure where the peptide lied in the CDRs similar to the X-ray crystal structure of HIV-1 p24 bound with antibody (PDB ID: 1AFV) was then selected.

### 2.2. Molecular Dynamics Simulation and Binding Free Energy Calculations

Energy minimization and MD simulations were performed using PMEMD.CUDA from AMBER12 [18] on GPUs Quadro 2000D produced by NVIDIA which speed up the simulation wall time required to obtain the trajectory files from each simulation. MD simulations were carried out at the molecular mechanics level using the AMBER03 force field. Antibody-peptide structures were solvated in a cubic box of TIP3P water extending at least 10 Å in each direction from the solute, and the cut-off distance was kept to 12 Å to compute the nonbonded interactions. All simulations were performed under periodic boundary conditions [19], and long-range electrostatics were treated by using the particle-mesh-Ewald method [20, 21]. The time step was set to 1 fs and the trajectory was recorded every 0.1 ps. Prior to MD simulations, the systems were relaxed by a series of steepest descent (SD) and conjugated gradient (CG) minimizations. MD simulations were performed based on each of the minimized systems by gradually heating over 60 ps from 0 to 310 K with the protein atoms fixed using a force constant of 5 kcal/mol/Å^2^. Then, a 200 ps pressure-constant period (NPT) was applied to obtain an equilibrated density of the constrained protein atoms. The following step was a 40 ps-volume-constant period (NVT) at a force constant of 2.5 kcal/mol/Å^2^ followed by 100 ps dynamics at a force constant of 1.25 kcal/mol/Å^2^. Finally, an unrestrained MD simulation (no force applied on any protein atoms) was performed for each fully flexible system in the NVT ensemble at a constant temperature of 310 K for a total simulation time of 10 ns. 500 snapshots were collected from the last 5 ns of MD simulations for binding free energy analysis. Equilibration was monitored by convergence in terms of the temperature, energy, and density of the system and the root-mean-squared deviations (RMSD) of the backbone atoms compared to the docking structure.

The binding free energies (Δ*G*_bind_) were evaluated according to the strategy described by Massova and Kollman [2]. Below it is summarized: the Δ*G*_bind_ was determined from the free energies of the complex, protein, and peptide according to the following equation:

(1) ΔGbind=ΔGwater(complex) −[ΔGwater(protein)+ΔGwater(peptide)].

Based on the selected MD snapshots, the binding free energy for each antibody-peptide system could be estimated using MM-PBSA [22]. The binding free energy, Δ*G*_bind_, is written as the sum of the gas phase contribution, Δ*G*_gas_, the desolvation free energy of the system upon binding, Δ*G*_desolv_, and an entropic contribution, −*T*Δ*S*, as seen in Figure 7, where the term Δ*H*_gas_ contains the van der Waals (Δ*E*_vdW_) and electrostatic (Δ*E*_elec_) interaction energies between the two partners in the complex and the internal energy variation (including bond, angle, and torsional angle energies) between the complex and the isolated molecules (Δ*E*_intra_), respectively; −*T*Δ*S* is the change of conformational entropy upon peptide binding, which is not considered here because of its high computational demand and relatively low accuracy of prediction [5]. All energies are averaged along the MD trajectories. Δ*G*_desolv_ is the difference between the solvation free energy, Δ*G*_solv_, of the complex and that of the isolated parts. Δ*G*_solv_ is divided into the electrostatic, Δ*G*_elec,solv_, and the nonpolar, Δ*G*_np,solv_, contributions: Δ*G*_solv_ = Δ*G*_elec,solv_ + Δ*G*_np,solv_.

For the MMPBSA calculations, Δ*G*_elec,solv_ was calculated with a built-in module, the PBSA program in AMBER12 which solves the Poisson-Boltzmann equation. The grid size for the PB calculations was 0.5 Å. The values of the interior and exterior dielectric constants were set to 1 and 80, respectively. Δ*G*_np,solv_ was estimated based on the solvent accessible surface area (SASA) as Δ*G*_np,solv_ = 0.0072 × SASA, using the MolSurf program. The scFv anti-p17/peptide interaction energy profiles were generated by decomposing the total binding free energies into residue-residue interaction pairs by the MM-GBSA decomposition process in the MM-PBSA program of AMBER12. The calculated binding energies herein were not absolute ones, since we do not include the entropic changes of the solute molecule. It is a computationally expensive to estimate entropic changes using normal mode analysis and the calculation tends to have a large error that introduces significant uncertainty in the result. Involving entropy in the calculation would not make much difference for the comparison of the binding free energies because of the similarity of the short peptides binding to the same scFv.

### 2.3. Computational Alanine Scanning

Alanine scanning [22], a computational method of systematic alanine substitution, has been particularly useful for the identification of functional epitopes. Substitution with alanine removes the side chain atoms of the residues in CDR loops of wild type scFv anti-p17. All the alanine mutant structures were obtained by deleting atoms and truncating the mutated residue in the hypervariable portions of the loops on the heavy chain (H1–H3) and light chain (L1–L3) of wild-type scFv anti-p17. All parameters in the topology files for the mutated residues were accordingly replaced by the alanine residue parameters. Proline residues were not mutated since their backbone conformations differ significantly from the alanine residue [2]. As a result, the Pro58 and Pro230, which belong to the CDR loops H2 and L3, respectively, are not selected. Then the modified parameter files were generated again by using the LEaP module [5]. This was extrapolated to the snapshots collected from the trajectories at the last 500 ps resulting from the MD simulations by using the script mm_pbsa.pl implemented in the AMBER package. From the decomposed energy and alanine scanning result, the point mutation has been selected and investigated.

### 2.4. Site-Directed Mutagenesis and the Evaluation of the Binding Activity of scFv Anti-p17

To generate scFv anti-p17 mutant, phagemid was subjected to perform mutation procedure following the instruction of site-directed mutagenesis (Stratagene). Briefly, ten nanograms of phagemid template were mixed with 125 ng of mutated primers in provided buffer. *Pfu*Turbo DNA polymerase (Stratagene; 2.5 U) was added to the mixture for cycle amplification. The reaction started with one round of 95°C for 30 s followed by 16 rounds consisting of 95°C for 30 s, 55°C for 1 min, and 68°C for 9 min. 10 U of *Dpn*I restriction enzyme (Stratagene) was subsequently added to eliminate phagemid template and incubated for 1 hour at 37°C. The reaction tube was subsequently placed on ice for 2 min. This synthesized product was further transformed into *E. coli* XL-1 Blue. Bacterial containing mutant phagemid was then cultured for production of phage-displayed mutant scFv anti-p17 as described elsewhere [6]. To evaluate the binding activity of wild type and mutant scFv anti-p17 with a series of synthetic peptides (GenScript, Piscataway, New Jersey, USA), phage ELISA was set up as described in our previous study [6].

## 3. Results and Discussion

### 3.1. Pairwise Decomposition Energies and Computational Alanine Scanning

**Figure 1:**
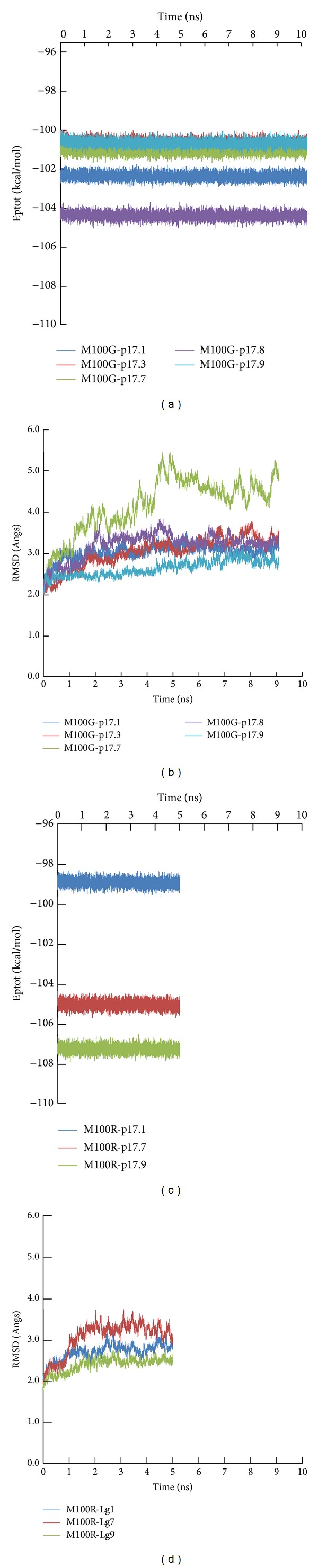
The potential energy (kcal/mol) and the root-mean-squared deviations (RMSD) of the backbone atoms compared to the docking structure for mutants (M100G, M100R) of scFv anti-p17 bound to its variants during the production run were monitored.

**Figure 2:**
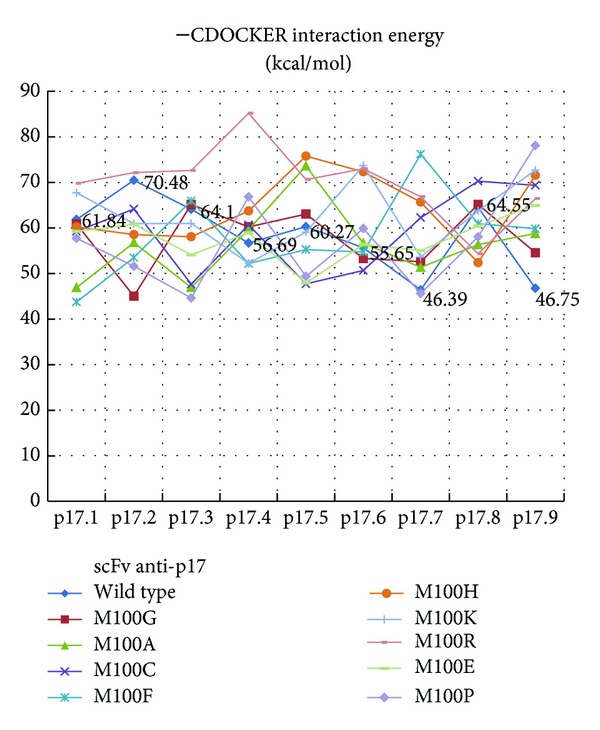
The –CDOCKER interaction energy (kcal/mol) of original scFv anti-p17 bound to its variants was compared with the different mutants from point mutation at MET100.

**Figure 3:**
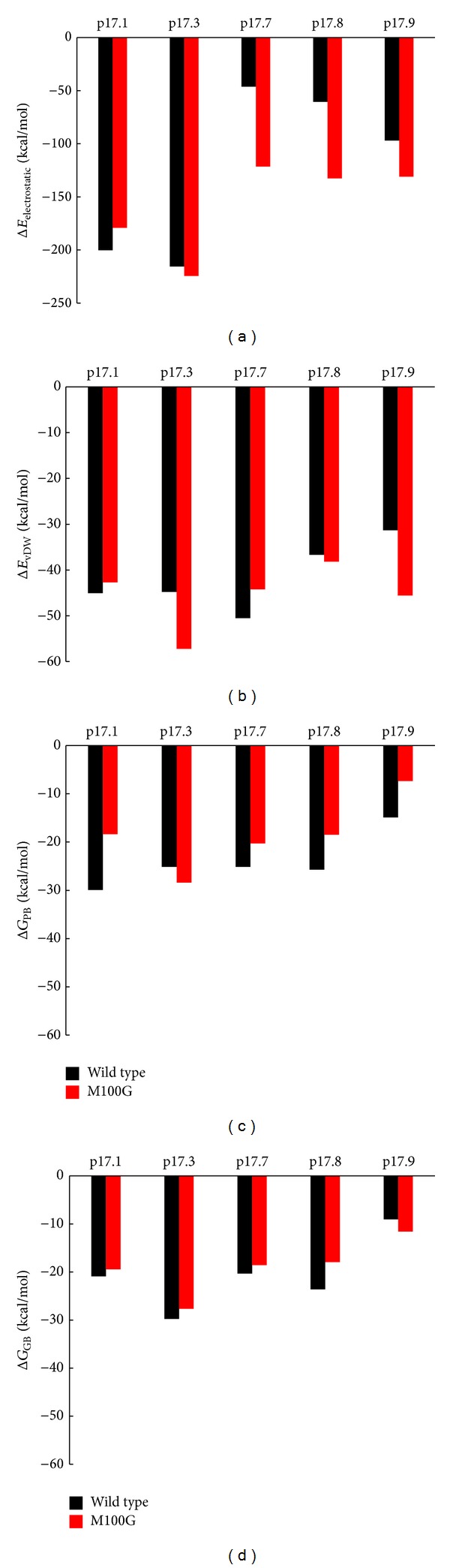
Decomposition energy of amino acids in the CDRs from 2 ns simulations with series of peptides 17.1, 17.3, 17.7, 17.8, and 17.9 for wild and mutant (M100G) type scFv anti-p17: (a) Δ*E*_electrostatic_; (b) Δ*E*_vdW_; (c) Δ*G*_PB_; (d) Δ*G*_GB_. A total of 500 snapshots were collected at 1 ps intervals from the last 500 ps of 2 ns MD for binding free energy analysis.

The comparison of experimental activities, peptide ELISA, with the results of CDOCKER interaction energy derived from molecular docking (CDOCKER) suggested that the experimental value had a high correlation (*r* ^2^ = 0.84) with the CDOCKER interaction energy (Figure 2). From Table 1, peptide p17.7 had the lowest score (less favorable interaction energy) and p17.8 had the highest score (more favorable interaction energy), whereas p17.3 had very similar score to that of the wild-type peptide. The peptide ELISA was used to describe the binding activity of scFv anti-p17 to its target peptide (p17.1) and four mutant peptides (p17.3, p17.7, p17.8, and p17.9). Positive signals were observed in all peptide coated wells, indicating that this scFv anti-p17 could bind to all mutant peptides. Peptide p17.8 gave the highest signal followed by p17.1, p17.3, p17.9, and p17.7, respectively. Although a good correlation between the docking scores and ELISA competitive binding activity was found, there is some difference between p17.8 and p17.3. It should be remarked here that only slightly difference between p17.8 and p17.3 was observed with a limitation of receptor rigidity in molecular docking in the screening process. Furthermore, we selected five peptide epitopes consisting of one wild-type peptide (p17.1) and four mutated peptides (p17.3, p17.7, p17.8, and p17.9) for further investigation by molecular dynamics simulations (MDs) and MM-PBSA. To validate the dynamic stability of the complexes, total potential energy and the RMSD for the backbone atoms along the 10 ns MD trajectories using the initial minimized docking structure as a reference were monitored in Figure 1. The RMSD values of most complexes converged around 2.5–3.5 Å, which means the MD trajectories of the complexes appear to be well equilibrated. Some systems of scFv anti-p17 mutant complexes were not stable and RMSD still did not converge after 10 ns. In order to investigate protein binding capability, the results derived from MM-PBSA and MM-GBSA calculations (Figures 3(c) and 3(d)) were compared and MM-PBSA shows higher correlation with experimental value. Therefore, the value of PBTOT was used to compare the simulation with the peptide ELISA results. The more negative the value, the more favorable the binding. The binding energies identified by the MM-PBSA protocol were ranked as follows: peptide p17.1 > p17.8 > p17.3~p17.7 > p17.9 with the values of −29.93, −25.70, −25.15, −25.15, and −14.88 kcal/mol, respectively. The major contributions to the binding free energy arise from the electrostatic energy, as calculated by the molecular mechanic (MM) force field (ELE), and from the electrostatic contribution to the solvation free energy, as calculated by PB (PB_CAL_); van der Waals contribution arises from MM (VDW). For the five binding peptides with the wild, type scFv, Δ*E*_vdW_, Δ*G*_PB_, and Δ*G*_GB_ are quite similar but the electrostatic energies were quite varied among the low and high activities peptides, indicating that the main factor determining the binding activity might arise from the electrostatic contribution as peptide p17.7 had a very low electrostatic contribution (−46.28 kcal/mol) compared to other sequences (Figure 3(a)).

**Figure 4:**
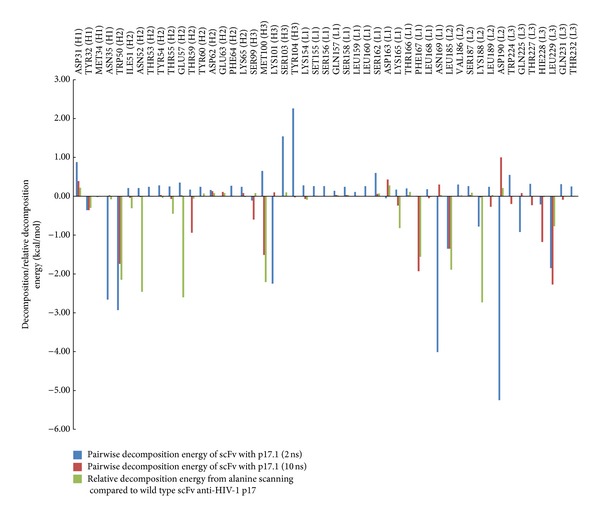
Histograms reporting the calculated pairwise decomposition energy which negative numbers in Δ*G*_bind_ mean highly favorable binding and the relative decomposition energy (Δ*G*_bind(ala)_ − Δ*G*_bind(wt)_) from the computational alanine scanning mutagenesis experiments compared to wild type of scFv with p17.1. The total bar height reflects the relative binding free energies of each amino acid in CDRs loops with wild type of scFv anti-p17 whose mutation to alanine by alanine scanning mutagenesis. The negative numbers indicated the preference for alanine mutation. Pairwise decomposition analysis 2 ns was extracted from 500 snapshots from 1.5–2 ns whereas the analysis 10 ns was from 500 snapshots from 5–10 ns simulations.

In order to elucidate the key residues in the binding pocket of the scFv anti-p17 and the most favorable interaction modes, in this study computational alanine scanning was performed. This method depends on the assumption that local changes of the protein do not influence the whole conformation of the complex significantly. In the calculation with alanine scanning, the positive and negative relative values in Figure 4 indicate the unfavorable and favorable for alanine substitution, respectively. All the residues in the hypervariable portions of the loops on the heavy chain (H1–H3) and light chain (L1–L3) of wild-type scFv anti-p17 have been mutated to alanine, except for the proline residue whose backbone is remarkably different from that of alanine. In comparison with wild type, the calculated binding free energy and computational alanine scanning analysis (Figure 4) demonstrated the importance of the residues of scFv anti-p17 in the binding pocket which are TRP50, ASN52, GLU57, MET100, LEU185, and LYS188 with the relative decomposed energy below −2.00 kcal/mol. Our previous results demonstrated that the specific residues located in the complementary determining regions (CDRs) of scFv anti-p17, MET100, LYS101, ASN169, HIS228, and LEU229 play a crucial role in the different binding affinities with the HIV-1 p17 variants [6]. Therefore, from both results the importance of the mutation's location has 10 positions in the CDRs of scFv anti-p17 consist of TRP50, ASN52, GLU57, MET100, LYS101, ASN169 LEU185, LYS188, HIS228, and LEU229. The methionine at position 100 (MET100) is initially selected for mutation due to the high contribution in binding energy of this region from position 100–104 residues and the substitution of alanine at this position showed the significant change in the binding interaction.

### 3.2. Structural Analysis of scFv Anti-p17 Point Mutations

**Figure 5:**
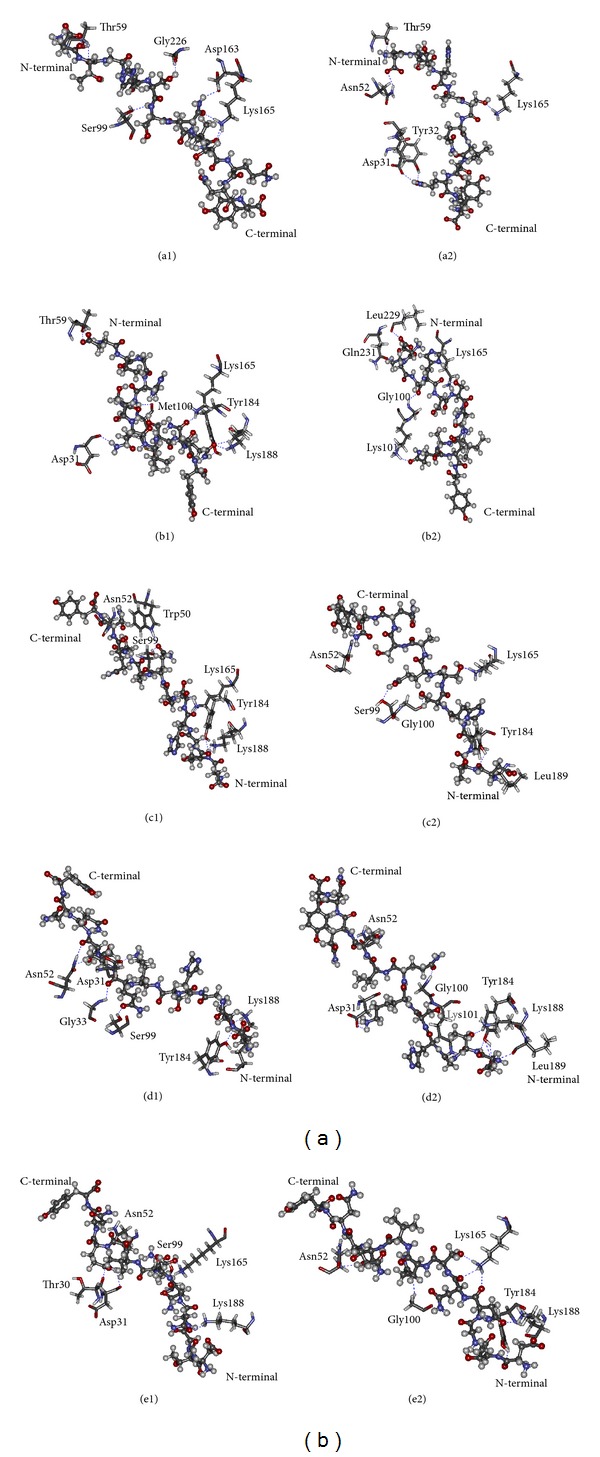
Docking structural comparison of wild type (1) and mutant form (M100G, 2) of scFv anti-p17 against five peptides: (a) p17.1, (b) p17.3, (c) p17.7, (d) p17.8, and (e) p17.9, respectively. A stick representation of side chains of scFv predicted to interact through hydrogen bond to peptides (scaled ball and stick). Hydrogen bonds are presented as dashed lines.

**Figure 6:**
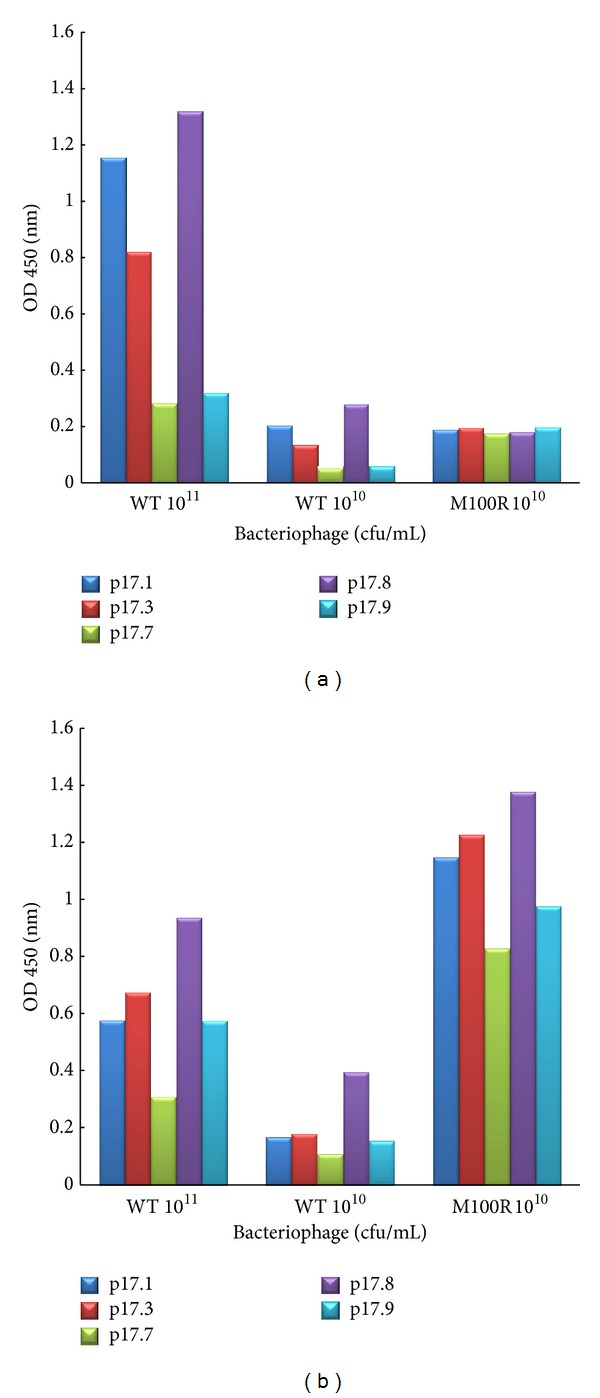
The indirect ELISA results of binding efficiency for scFv anti-p17 wild type and selected mutation M100R (a) and M100G (b). The values shown here are the results from second experiments.

**Figure 7:**
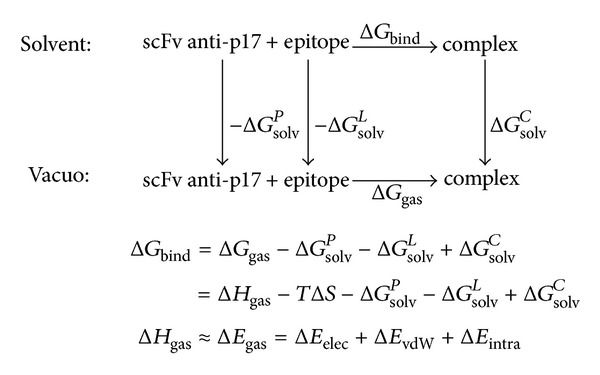


With the combination of pairwise decomposition energies and computational alanine scanning, the key amino acids in binding, TRP50, ASN52, GLU57, MET100, LYS101, ASN169, LEU185, LYS188, HIS228, and LEU229, were the important residues to binding efficiency of natural HIV epitope at the C-terminal on p17. Therefore, these residues are subjected to mutation in order to improve the binding activities. The residues roughly equivalent were grouped together in five subsets according to statistically preferred substitutions which generally correlate with side chain physicochemical properties, observed in buried residues and exposed residues reported by Bordo and Argos [15] as follows: (a) buried in the protein core (solvent-accessible surface for both residues ≤ 10 Å^2^), (b) exposed (solvent-accessible surface area ≥ 30 Å^2^), and (c) all the possible accessibility states allowed. The mutation of amino acids in the CDRs of scFv anti-p17 has been initially selected at position M100 since the pairwise decomposed energy indicated the unfavorable residue interaction and the substitution of the residue with alanine can lead to significant change in binding affinity. Mutation of methionine residues in position 100 of scFv anti-p17 (wild type) was mutated to another polarity group with electrically charged or uncharged or hydrophobic side chains which have the impact on the development of protein cores in structures maintaining main-chain fold [23] according to Table 1. Finally, the mutated scFv anti-p17 was docked with nine peptides by CDOCKER. The energies obtained from the docking of each peptide with the mutated scFv are listed in Table 1. The more negative interaction energies exhibit the more favorable binding. The prediction of interaction energy with most peptides and mutant forms (M100G) is more than that of wild type scFv anti-p17 and the improved electrostatic contribution was observed as in Figure 3(a) for the mutant type. The hydrogen bond interactions of mutant form of scFv anti-p17 with five peptides at the residues ASP31, TYR32, ASN52, THR59, SER99, GLY100, LYS101, LYS165, TYR184, LYS188, LEU189, LEU229, and GLN231 were shown in Figure 5. Both of the two peptides (p17.1 and p17.3) showed interactions with the receptor mostly at TYR32, THR59, and LYS165, and other three peptides (p17.7, p17.8, and p17.9) showed interactions with the receptor mostly at ASN52, GLY100, and TYR184. From the conformation with the lowest CDOCKER interaction energy structure of M100G scFv anti-p17, the peptides bound in two orientations, where the N-terminal (p17.1–p17.6) and the C-terminal (p17.7–p17.9) of peptide sequences were directed toward the binding pocket. The calculated docking interaction energy between single mutation from methionine 100 to glycine (M100G) and peptide sequences, p17.1 (DTGHSSQVSQNY), p17.3 (DTGHSSQISQNY), p17.7 (DTGHSSQASQNY), p17.8 (DTGHSKQVSQNY), p17.9 (DTGNNSQVSQNY), has shown the favorably interaction energy compared to wild type which correlated well with the indirect ELISA (Figure 6). Detail on experimental results will be discussed in the following section.

### 3.3. Comparison of Calculated Binding Free Energy with Experimental Data

From the indirect ELISA results in Figure 6, the different signal from the same number of added phage particle (10^11^ and 10^10^ CFU/ml) was observed. We identified p17.7 and p17.9 as the low affinity binding peptides, whereas the p17.1, p17.3, and p17.8 were identified as the high affinity binding peptides with our scFv wild type. In the same figure, the signal of phage displaying scFv anti-p17 mutant (M100R and M100G) was higher than that of scFv wild type reflected the higher binding activity suggested that both mutants can improve the binding affinity with the peptide. However, M100G in phage 10^10^ CFU/ml shows significantly improvement in binding affinity a lot more than M100R. The binding efficiencies were ranked as follows: peptide p17.8 > p17.1 > p17.3 > p17.9 > p17.7 for M100G. However, the energy calculation from CDOCKER did not explain very well why M100G is better than M100R. According to amino acids characteristics and structures for our residue mutation, arginine has the long side chain with the electrically positive charged amino groups; glycine is considered small nonpolar side chain and weakly hydrophobic, where methionine as in the scFv wild type has larger side chains and is more strongly hydrophobic. Possibly, the primary effect of arginine at M100R might be due to the disruption of the assembly between *V*_*H*_ and *V*_*L*_, as it causes the loss of important hydrogen bonds mediated by the M100R side chain, including a conserved interface hydrogen bond. Comparison of the complex stability was monitored from RMSD in Figure 1. We have observed several instable complexes of peptides p17.3 and p17.8 with M100R and cannot process for MD simulations. From Figure 3, the electrostatic contributions have been significantly improved with series of peptides 17.1, 17.3, 17.7, 17.8, and 17.9 for mutant (M100G) compared to wild-type scFv-p17 while other parameters such as Δ*E*_vdW_, Δ*G*_PB_, and Δ*G*_GB_ did not show much variation.

## 4. Conclusion

The identification of the key residues of scFv in the complementarity determining regions (CDRs) from the combination of the computational alanine scanning and pairwise decomposition energy calculation can be used to design the new potential scFv anti-p17. From the result, the importance of the residues which highly effect by alanine scanning of scFv anti-p17 are TRP50, ASN52, GLU57, MET100, LEU185, and LYS188 whereas from pairwise decomposition energy calculation, MET100, LYS101, ASN169, HIS228, and LEU229, play a crucial role in the different binding affinities with the HIV-1 p17 variants. The new antibodies were designed by mutating the potential amino acid residues in CDRs of scFv anti-p17. With the guide from both methods, the key residue at MET100 was initially selected to a single point mutation. The fast protocol of docking interaction energies can be used to estimate the binding affinity of the new scFvs with the series of natural peptides. The electrostatic contributions have been a major part in the antibody design while other parameters such as Δ*E*_vdW_, Δ*G*_PB_, and Δ*G*_GB_ did not show much variation. Long time scale MD simulations can monitor the stability of the novel scFv anti-p17 complexes. Concern on the disruption of the scFv which affects the binding activity due to the mutation is subject to further investigation. Peptide ELISA results confirmed the improved binding affinity of novel scFv anti-p17 mutants from the theoretical calculations.

## Figures and Tables

**Table 1:** –CDOCKER interaction energy (kcal/mol) of mutant (M100G) scFv anti-p17 bound to its variants.

Antibody	–CDOCKER interaction energy								
	p17.1	p17.2	p17.3	p17.4	p17.5	p17.6	p17.7	p17.8	p17.9
Competitive ELISA: percentage inhibition values (%) Mean ± STDEV	(1) 75.9 (2) 90.1 83.0 ± 10.0	—	55.2 59.6 57.4 ± 3.1	—	—	—	44.6 60.1 52.4 ± 11.0	79.5 87.6 83.6 ± 5.7	— 88.5 88.5 ± 0.0
scFv anti-p17 wild type	61.84	70.48	64.10	56.69	60.27	55.65	46.39	64.55	46.75
M100G	60.86	45.02	**65.16**	**60.23**	**63.05**	53.29	**52.59**	**65.16**	**54.55**
M100A	46.89	56.79	46.92	**59.62**	**73.61**	**56.66**	**51.36**	56.33	**58.78**
M100C	59.26	64.16	47.63	**60.66**	47.72	50.67	**62.26**	**70.26**	**69.37**
M100F	43.75	53.51	**65.93**	52.19	55.25	54.70	**76.20**	60.93	**59.83**
M100H	60.23	58.55	58.07	**63.74**	**75.78**	**72.33**	**65.68**	52.38	**71.55**
M100K	**67.72**	60.93	60.99	52.23	59.08	**73.71**	**53.63**	63.75	**72.68**
M100R	**69.78**	**72.14**	**72.64**	**85.19**	**70.63**	**72.99**	**66.85**	54.33	**66.46**
M100E	59.24	60.82	54.07	**58.95**	47.89	**56.39**	**55.02**	60.42	**64.95**
M100P	57.72	51.58	44.61	**66.83**	49.35	**59.87**	45.63	58.04	**78.10**

Note: Highlight in bold letter indicates the lower interaction energy than original scFv. Sequences of p17.1–p17.9 are ^121^DTGHSSQVSQNY^132^, ^121^DTGHSNQVSQNY^132^, ^121^DTGHSSQISQNY^132^, ^121^DTGHNSQVSQNY^132^, ^121^NTGHSSQVSQNY^132^, ^121^DTGNSSQVSQNY^132^, ^121^DTGHSSQASQNY^132^, ^121^DTGHSKQVSQNY^132^, and ^121^DTGNNSQVSQNY^132^, respectively.
